# Supplementary figures and images for: Optimizing network propagation for multi-omics data integration
Source: PLoS Comput Biol. 2021 Nov 11;17(11):e1009161. doi: 10.1371/journal.pcbi.1009161 (PMC8664198; doi:10.1371/journal.pcbi.1009161)

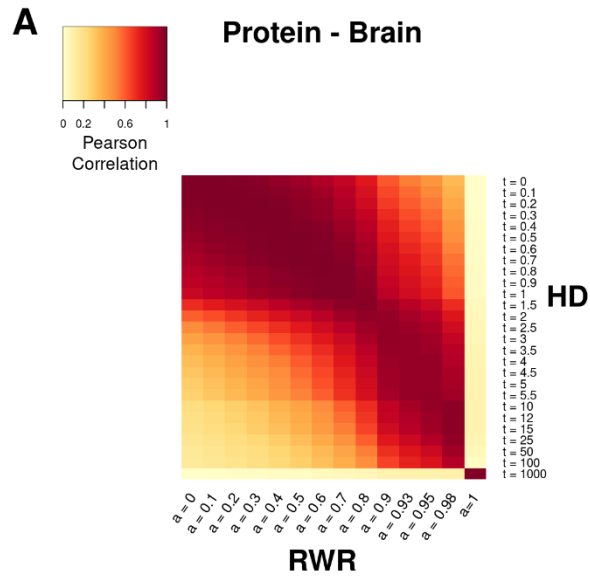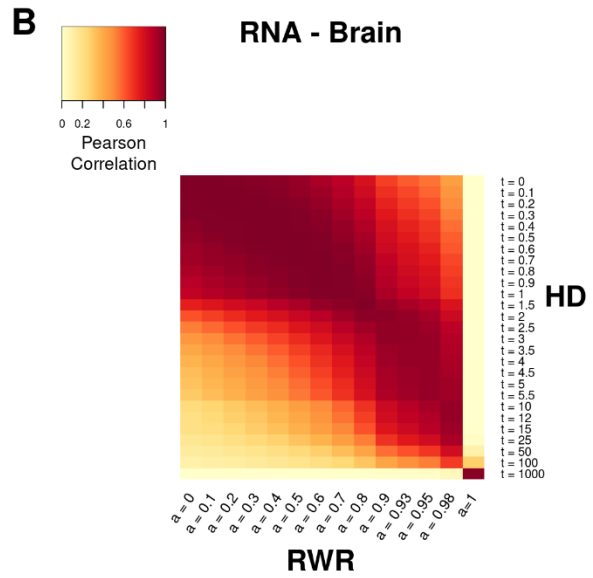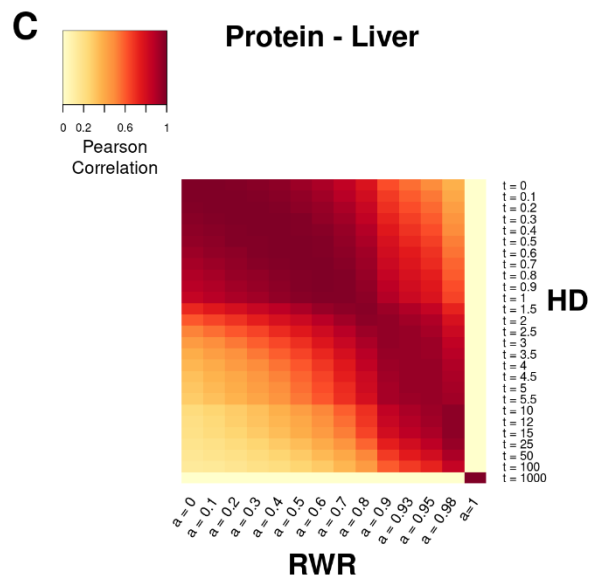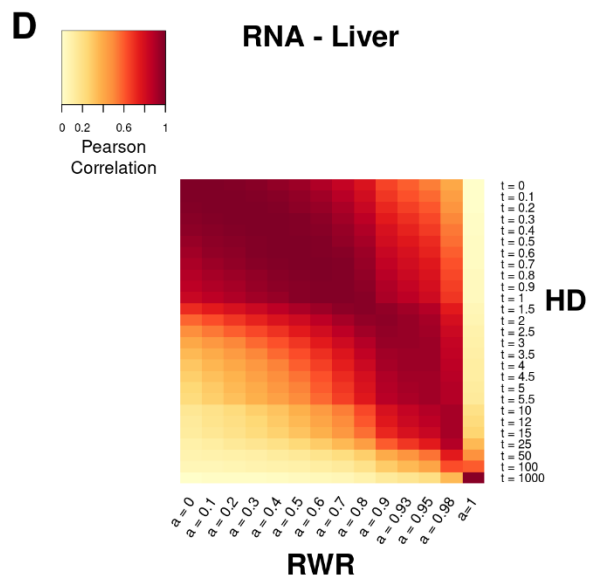

Supplement: S1 Fig — The smoothed log fold changes for proteome of brain (A) and liver (C) tissue and transcriptome of brain (B) and liver (D) tissue computed with RWR (varying α between 0 and 1) and HD (varying t between 0 and 1,000) are correlated. For the analysis, all genes which are present in the network (9,388 genes) are included. (PDF) [file pcbi.1009161.s001.pdf]

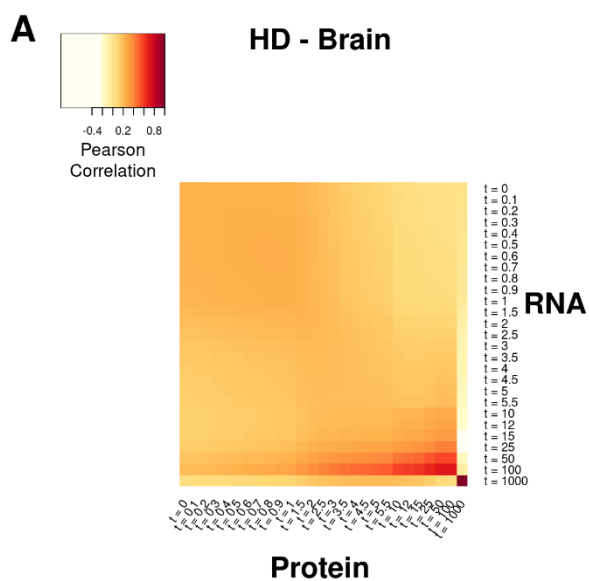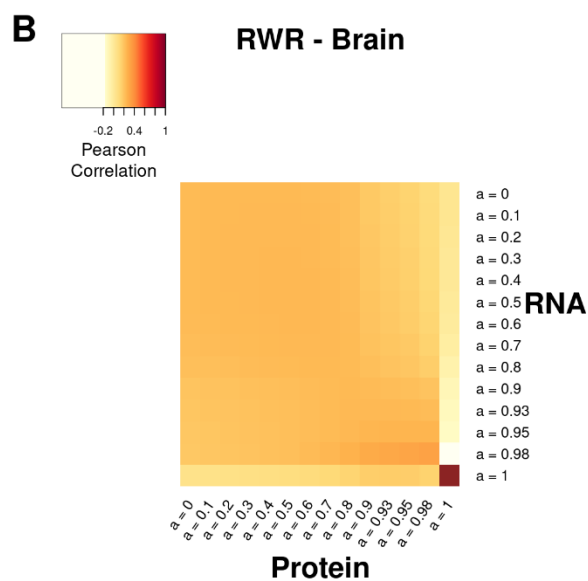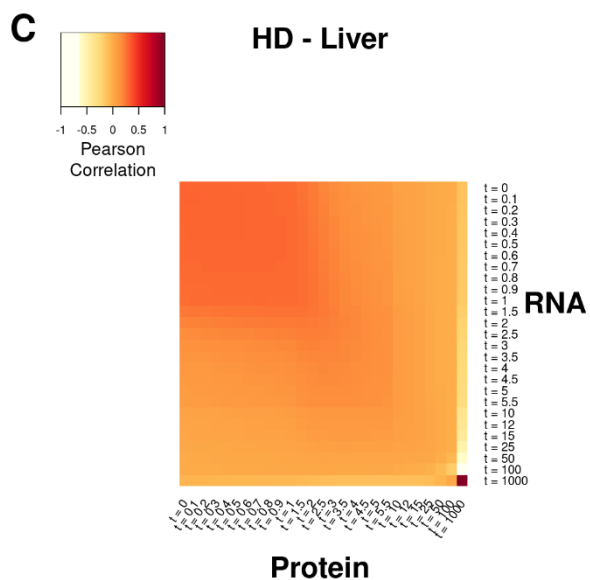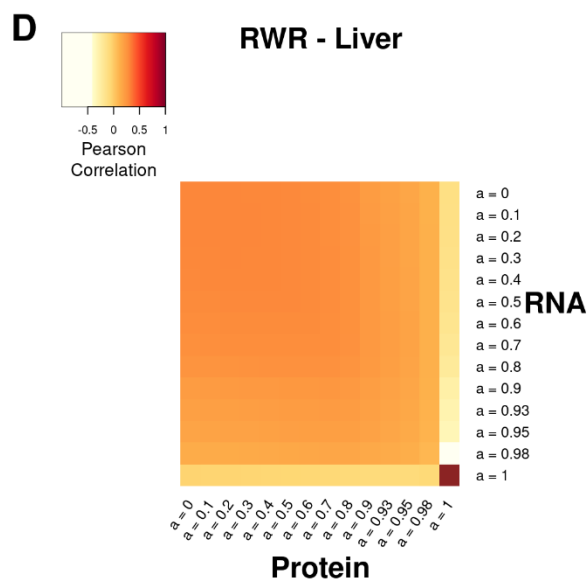

Supplement: S2 Fig — For brain tissue the correlation between mRNA and protein levels of ageing tissues during network propagation increases with both algorithms, HD (A) and RWR (B). Whereas for liver the consistency cannot be improved using network propagation with HD (C) or RWR (D). In the analysis, all genes which are present in the network as well as expressed and quantified with RNA-Sequencing and MS proteomics (n = 1,772 for brain, n = 1, 670 for liver) are included. (PDF) [file pcbi.1009161.s002.pdf]

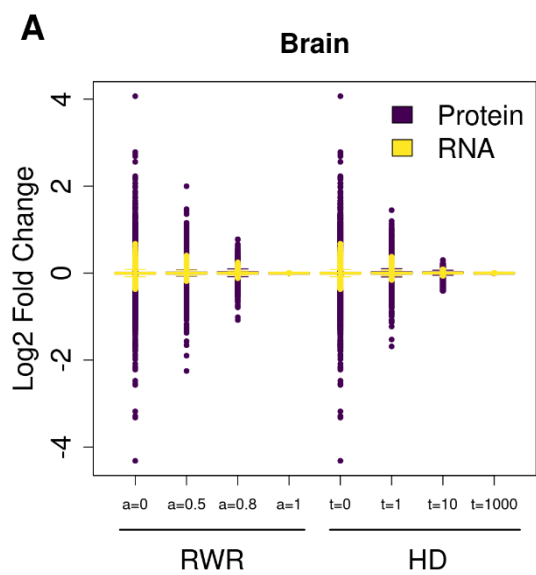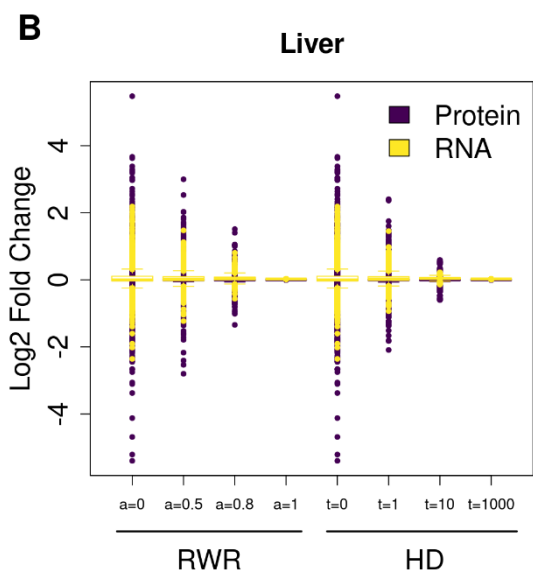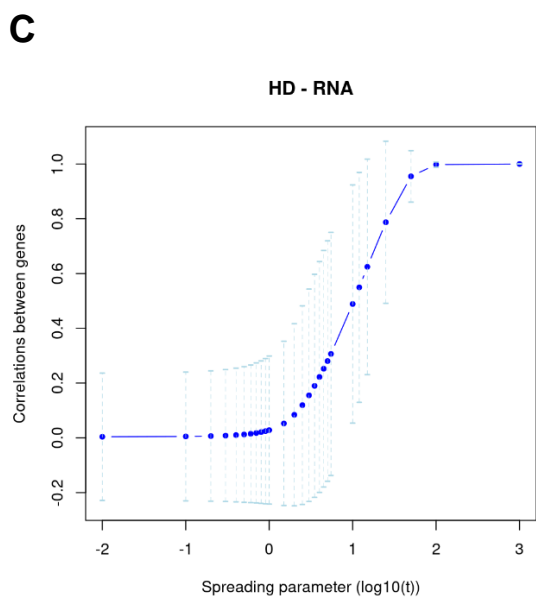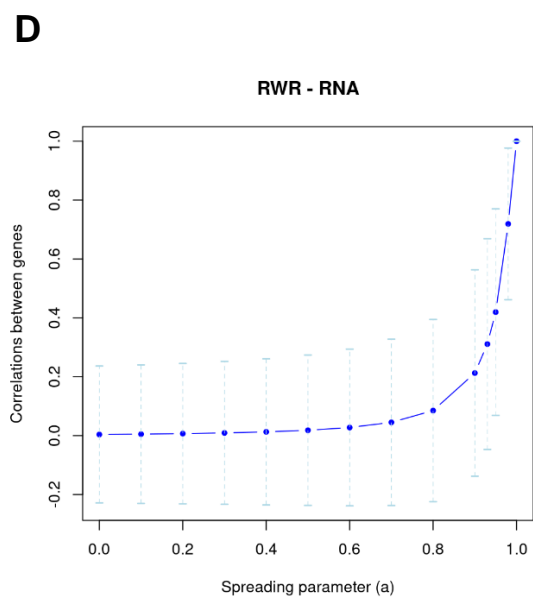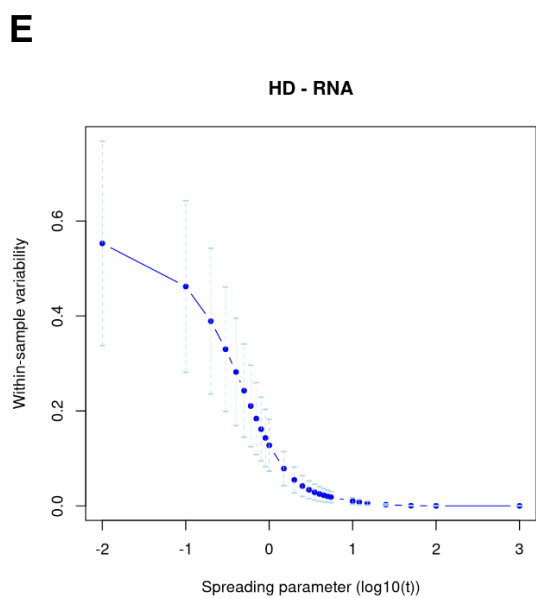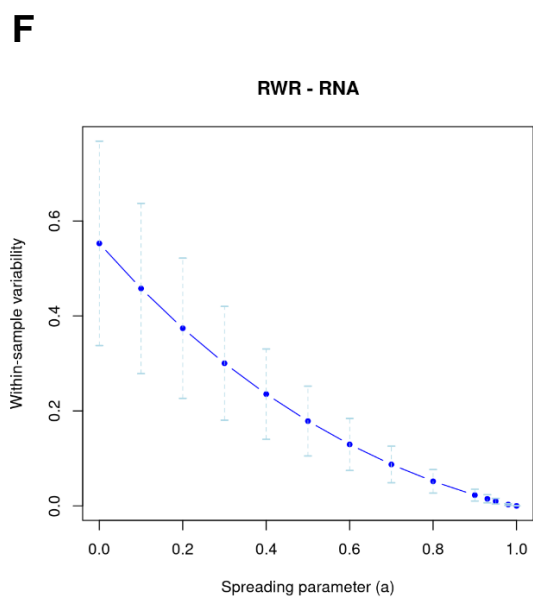

Supplement: S3 Fig — A, B: The boxplots show the distributions of protein and RNA log2 fold changes for the brain (A) and liver (B) from Ori et al. [13] dataset before propagation (ɑ = 0, t = 0) and after propagation with RWR and HD using varying parameters (ɑ = 0.5, 0.8, 1 and t = 1, 10, 1,000). C, D: For each value of the spreading parameter, all pairwise correlations between genes across samples were computed using the corresponding propagated mRNA log2 fold changes of the PCa study with the two propagation algorithms (RWR and HD). Subsequently, the average pairwise correlation and standard deviation (SD; across the gene pairs) was computed for each value of the spreading parameter. Average pairwise correlation across the values of the spreading parameter with HD (C) and RWR (D) (blue curves). Error bars indicate the average +/- standard deviation. E, F: For each value of the spreading parameter, we computed for each PCa tumor sample the variance across its propagated mRNA log2 fold changes (within-sample variability) with the two propagation algorithms. Subsequently, the average within-sample variability and SD (across the tumor samples) was computed for each value of the spreading parameter. Average within-sample variability across the values of the spreading parameter with HD (E) and RWR (F) (blue curves). Error bars have been added in the same way. In the above analyses, we used the measured network nodes only to compute the pairwise correlations and the within-sample variability. (PDF) [file pcbi.1009161.s003.pdf]

**A**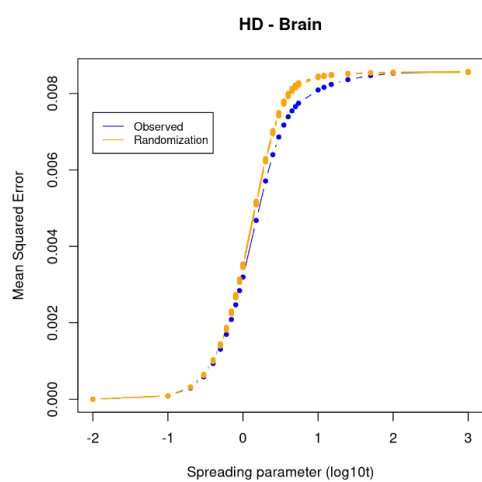**B**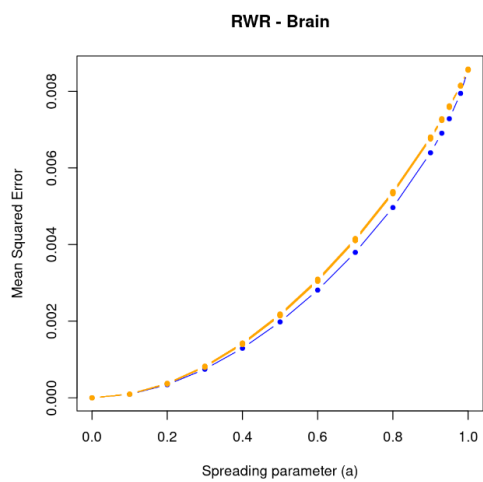**C**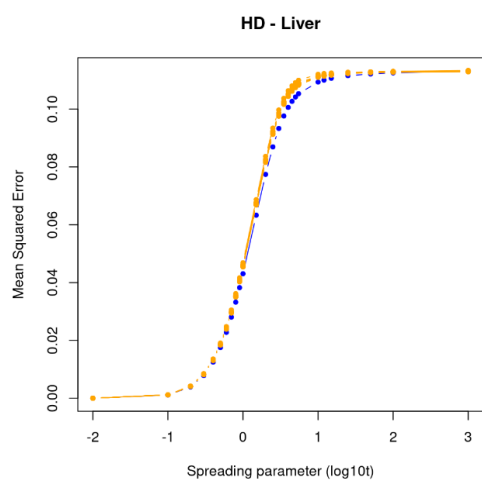**D**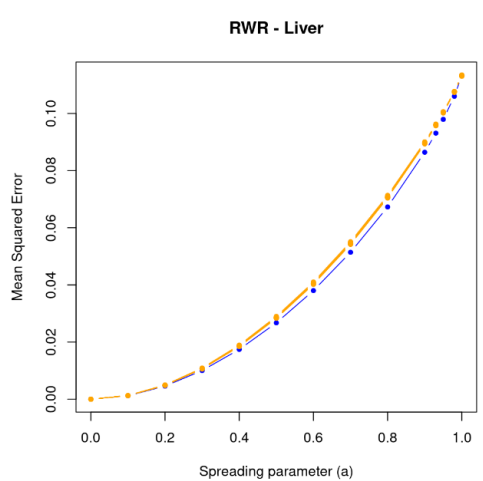**E**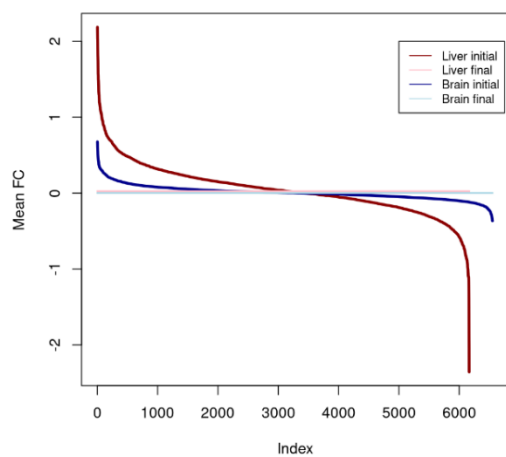

Supplement: S4 Fig — Observed bias2 curves (blue) (as defined in Material and Methods) across the values of the spreading parameter for the brain (A, B) and liver (C, D) using HD (A, C) and RWR (B, D). We also applied the following randomization approach: we permuted the average log2 fold changes of the transcriptome for the brain and liver and computed bias2 curves based on the permuted vectors. Bias2 curves are shown for 10 permutations (orange). The computation of the bias2 was based on the measured network nodes only. For the randomization approach, we only permuted the measured network nodes while the value of the unmeasured network nodes was always initially set to 0. E: Average log2 fold changes (of the measured network nodes) of the transcriptome for the brain (initial: dark blue, final (RWR, ɑ = 1): light blue) and liver (initial: dark red, final (RWR, ɑ = 1): pink) sorted in decreasing order. (PDF) [file pcbi.1009161.s004.pdf]

**A**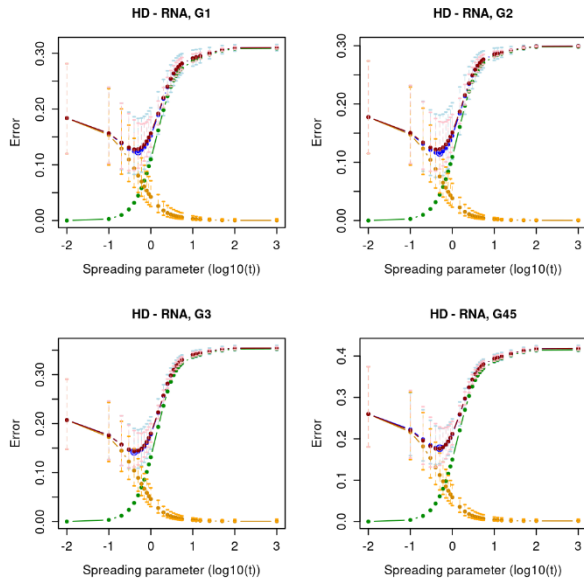**B**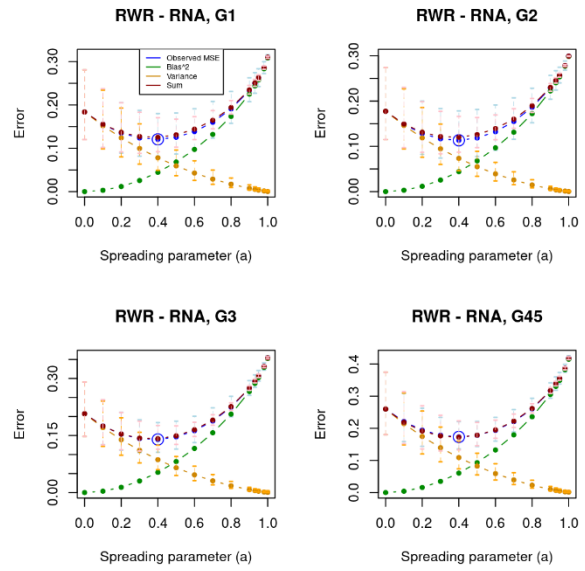**C**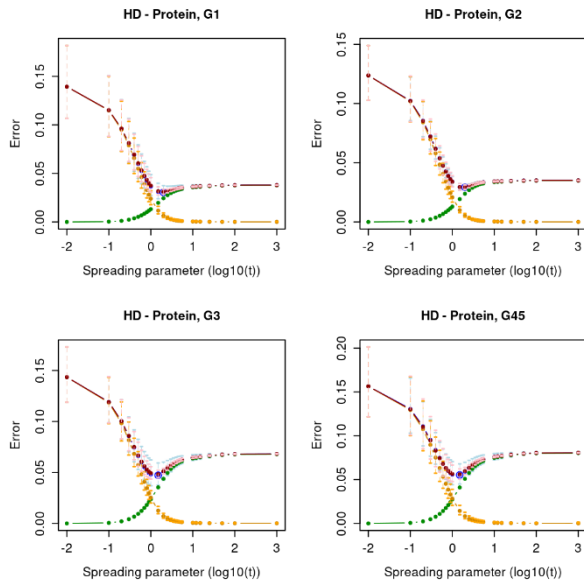**D**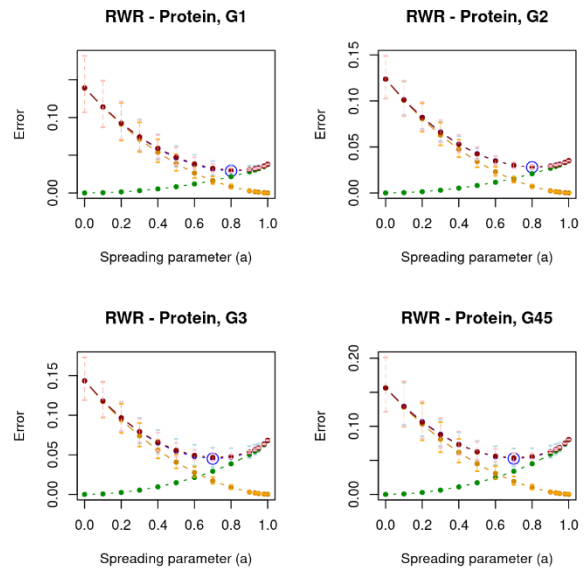

Supplement: S5 Fig — MSE curve (and corresponding bias-variance decomposition) across the values of the spreading parameter (similar to Fig 2) for each grade group separately: G1 (upper left), G2 (upper right), G3 (lower left) and G4/5 (lower right) for the mRNA with HD (A) and RWR (B) as well as for the protein layer with HD (C) and RWR (D). (PDF) [file pcbi.1009161.s005.pdf]

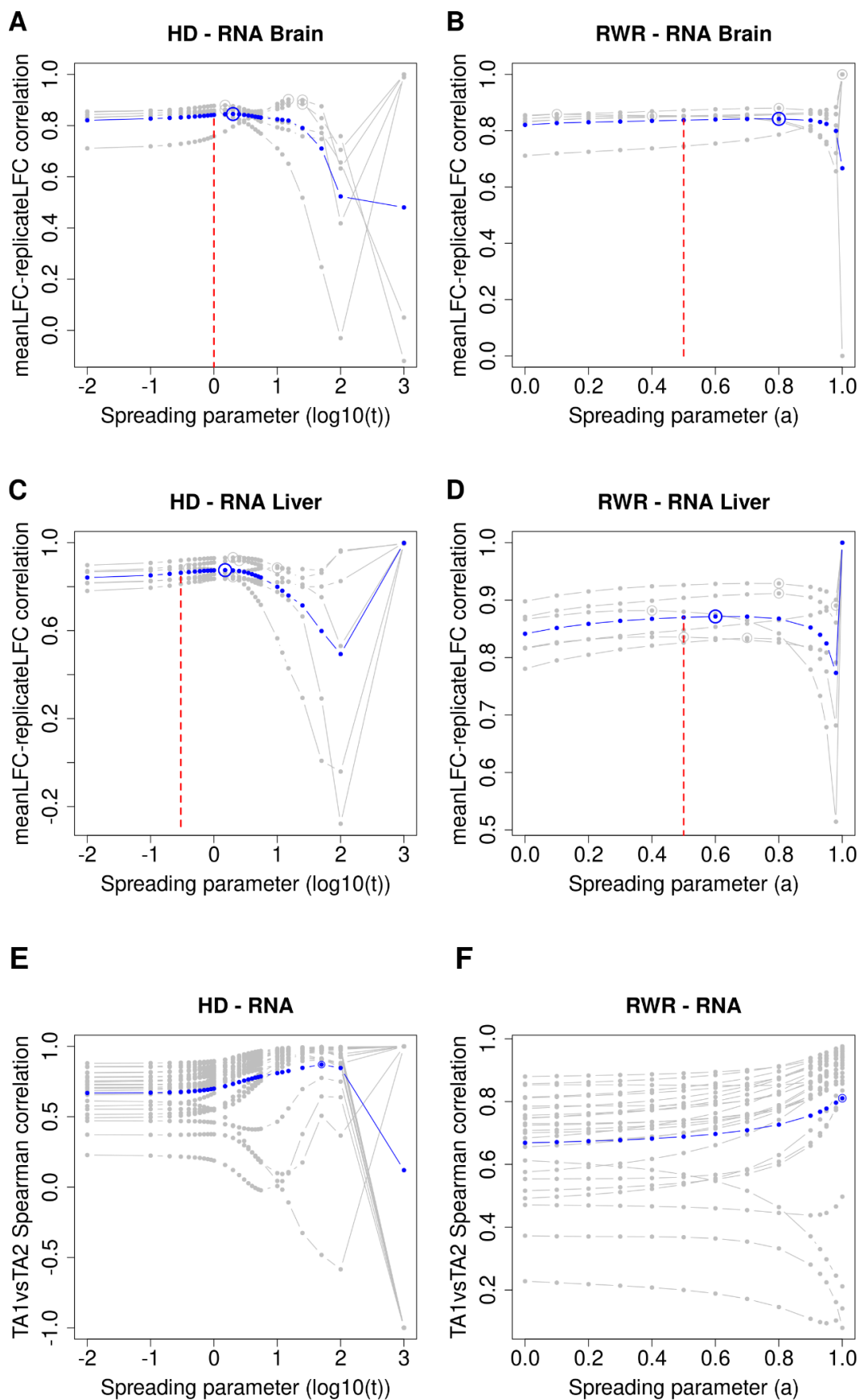

Supplement: S6 Fig — A, B, C, D, E, F: Same as Fig 4 but using Spearman correlation in the place of Pearson. (PDF) [file pcbi.1009161.s006.pdf]

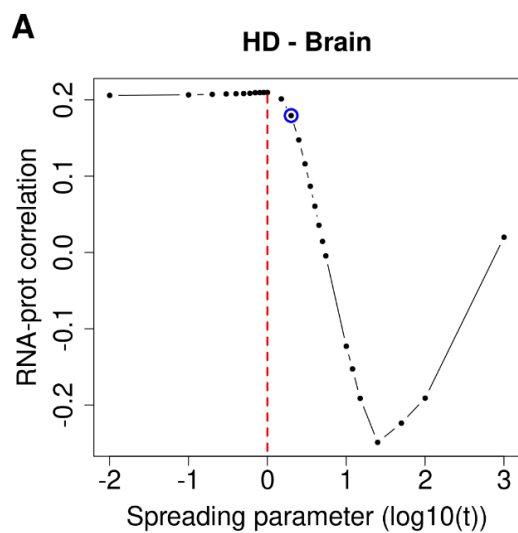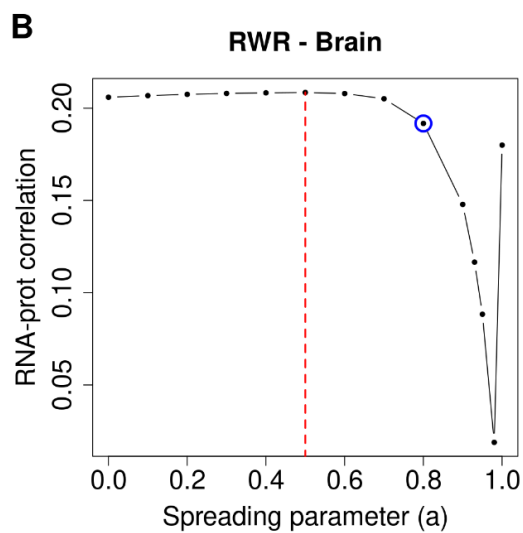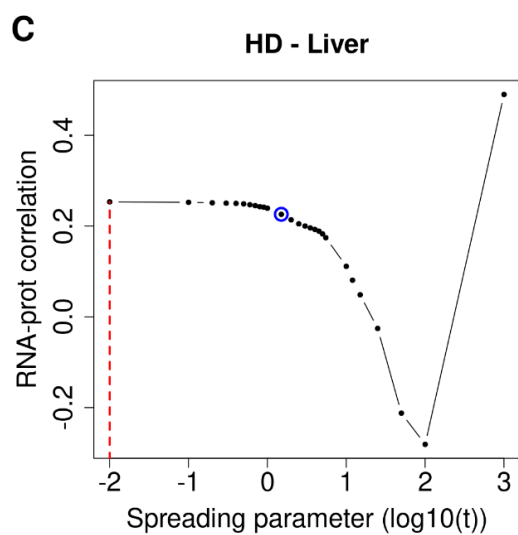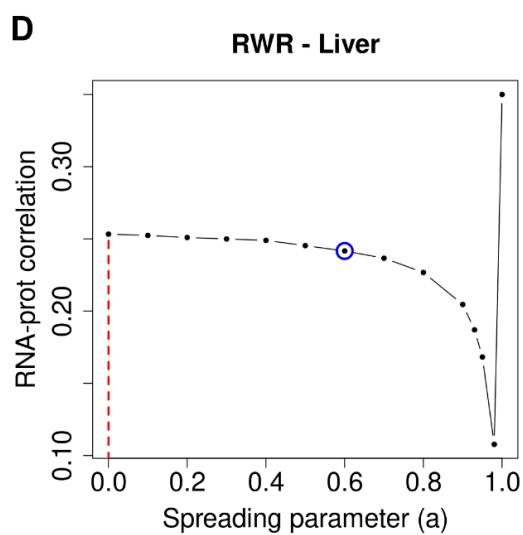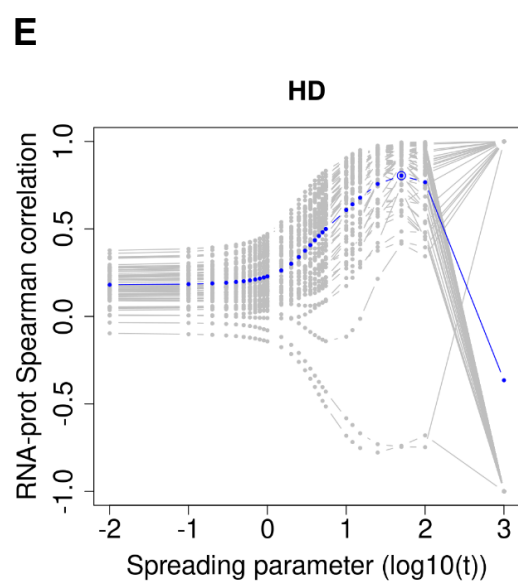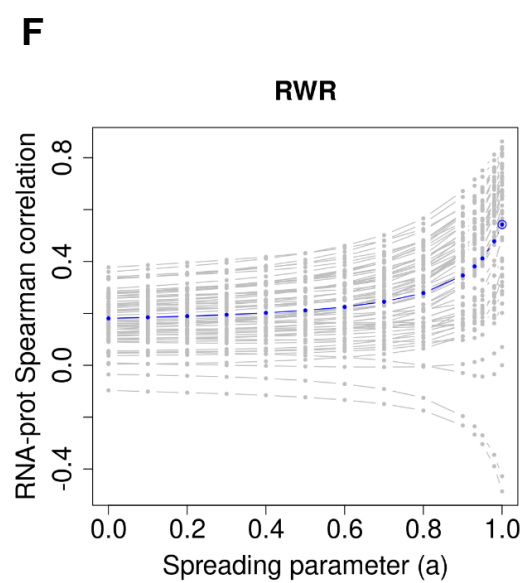

Supplement: S7 Fig — A, B, C, D, E, F: Same as Fig 5 but using Spearman correlation in the place of Pearson. (PDF) [file pcbi.1009161.s007.pdf]
