# Supplementary material for: Optimizing network propagation for multi-omics data integration
Source: PLoS Comput Biol. 2021 Nov 11;17(11):e1009161. doi: 10.1371/journal.pcbi.1009161 (PMC8664198; doi:10.1371/journal.pcbi.1009161)
Supplement: Attachment — Submitted filename: Response_to_reviewers.pdf [file pcbi.1009161.s009.pdf]

Dear Teresa Przytycka,

Thank you very much for subjecting our submission to peer review and for inviting a revised version of the manuscript! We are grateful to the two reviewers who have carefully read the manuscript and provided constructive feedback that clearly helped us to improve our submission.

We have addressed all of the reviewers' concerns (see below). Apart from text improvement, we have added two entirely new analyses that were both inspired by the reviewers' comments. First, we have added an assessment of gene-specific factors explaining the 'success' of smoothing, i.e. addressing the question: which genes benefit more from network propagation than others? (see new Fig. 3) Second, we evaluated how additional noise on the data affects the outcome. In particular we wanted to know if network propagation is capable of recovering the original signal before adding the noise (Fig. 2E-F).

We believe we could address all of the concerns and hope that you agree this manuscript can now be published in PLoS Computational Biology.

We thank you and the reviewers for your continued efforts and we are looking forward to your reply!

Andreas Beyer

In the following, Reviewer comments are marked in *blue italic*. Page numbers refer to the document without change tracking (but they are mostly the same in both versions).

# Reviewer #1

*The paper is a pleasure to read. It studies the algorithm of network propagation and suggests methods to normalize its scores and optimize its parameters. It is well structured and provides all necessary algorithmic details.*

We very much thank the reviewer for this encouraging feedback.

*1. However, it seems to neglect previous literature on this subject which is critical to its assessment. Specifically, there have been several previous attempts for normalization of propagation scores to derive non-biased or even statistical scores. It is essential to compare the paper's results to those methods to determine the performance of the currently suggested method (row normalization). For a recent reference that reviews also earlier work in this regard see*

*"NetCore: a network propagation approach using node coreness", NAR 2020.*

We would like to thank very much the reviewer for bringing this very interesting paper to our attention.

First, we would like to clarify that we are not suggesting any specific normalization method as a 'best' method. In the first part of our submission we are comparing different normalization methods and the main take home message is that some methods may lead to an unintended topology bias. We do not know whether or not a topology bias is always problematic for addressing all possible research questions. Our point is simply to raise the awareness for such a bias to prevent unintended artifacts. Subsequently, we are using the degree row-normalized adjacency matrix, simply because it would have been overly

complex to perform all subsequent analyses with multiple normalization methods. That however, does not imply that we think this is the sole best normalization method. In order to clarify this point, we have added the following statement at the end of that section:

"For the sake of simplicity we have decided to use this normalization method for the remaining parts of this publication. However, we do not intend to imply that the degree row-normalized adjacency matrix would be the sole possible choice. In fact, many other graph normalization methods have been proposed that might be suitable for a specific application of network propagation (Barel and Herwig 2020, Vanunu et al. 2010)."

This point is also related to Comment 7 of Reviewer #2; please see below.

In the NetCore paper, the authors suggest three normalization methods that involve the core of the nodes to address the node degree bias in PPI networks. Since the authors have shown that the normalization based on coreness alone is the most effective choice among the three (Fig. 4A) we focused next on this particular choice for the matrix *W*. To check whether this normalization leads to a topology bias, we computed for the rat network the propagated results for an input vector of 1s using the corresponding coreness-based matrix *W*. The result (similar to Fig. 1 in the paper) is shown next:

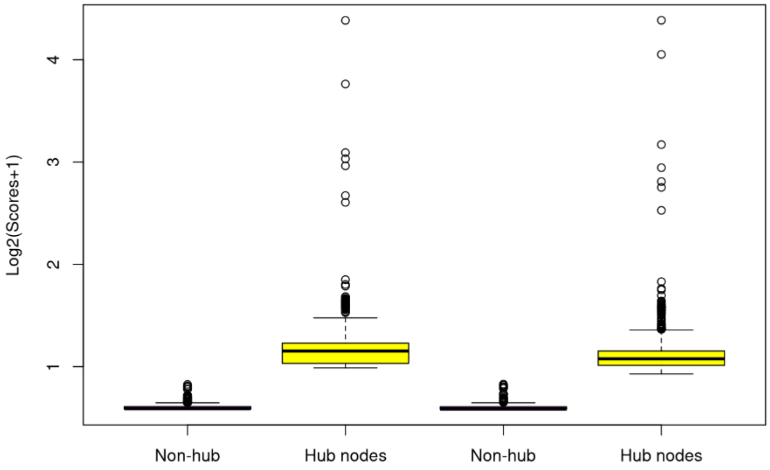


Fig: **Coreness based normalization induces a topology bias.** Distributions of log-transformed node scores after network propagation using the normalization based on coreness alone (t = 0.7 for Heat Diffusion and α = 0.5 for Random Walk with Restart). The input vector was a unit vector, i.e. all nodes had identical initial scores. Hub nodes gain higher average scores, whereas non-hub nodes get lower average scores.

Thus, we observe that the coreness-based normalization leads to a topology bias for both RWR and HD. We think that the suggested approach does not fully eliminate the node degree bias for the following reasons:

- Node degree and coreness are correlated as also shown in the paper. In this way, low-degree nodes will also have low coreness scores (e.g. nodes with one neighbor alone will have coreness equal to 1) while high-degree nodes will tend to have higher coreness scores.
- Further, for high-degree nodes with low coreness (like the hub nodes), these nodes although they have smaller probability to be visited from a node they still have many ways/neighbors to be

visited from and are thus expected to be visited more often throughout the random walk with restart.

Finally, for alpha=1 the *i*-th node score will be proportional to

$$k_i \sum_{j \text{ being neighbor of } i} k_j$$

where $k_i$ denotes the coreness of node *i*. From this formula, it is also obvious that the final score will depend on the node coreness (and thus the node degree) as well as the corenesses of its neighbors (and thus of the number of neighbors).

*2. Topology bias in my mind should be defined with respect to an empty prior which also coincides with setting alpha=1.*

We already have the following sentence in the paper on p. 7: "Note, that this is equivalent to the expected outcome for an infinite number of random input vectors with mean 1." To make this point more explicit, we have added the following sentence in the Methods section on p. 31:

"Equations (11)-(13) also hold true in terms of expectation (since the operations involve linear combinations): if the population mean vector $\mu$ was the unit vector (i.e. all genes initially had the same mean value) then the propagated mean vector would also be the unit vector or in other words the bias curve would be constant and equal to 0."

*3. In figure 1, can the authors compute the significance of the difference between hubs and non-hubs?*

The p-value from the two-sided Wilcoxon rank sum test is below $2.2 \cdot 10^{-16}$ (***) for both RWR and HD. We have added this information in the figure caption.

*4. It is mentioned that previous studies have chosen alpha arbitrarily but I don't think this is the case - rather performance for different alpha values was assessed.*

Many existing publications only report results for a single spreading parameter and do not explain (certainly not in detail) how those parameters were chosen. However, we agree with the reviewer: this does not mean that parameters were not carefully tested. Thus, we agree with the reviewer that we need to tone down our statement. For this reason, we have changed it to the following:

"In existing publications it is sometimes unclear to what extent different spreading parameters were tested and based on which criteria final parameters were selected."

*5. The limitations of the parameter selection method - e.g. having data with several replicates - should be clearly stated.*

We agree and we have added the limitations of the criteria in the Results section and in the Discussion (p. 26), which are the following: a) For computing the MSE and the inter-replicate consistency criterion (Pearson correlation) we need to have data with several replicates (as mentioned by the reviewer). For the between-dataset consistency we need to have multi-omics data with at least one sample in each available layer. Further, it must be biologically plausible that the node scores of the two layers are

supposed to be congruent. b) Selecting the optimal value based on the MSE was clearer compared to the Pearson correlation which sometimes needed a more careful inspection of the resulting curve.

## Reviewer #2

*Network diffusion approaches are commonly used for a variety of applications in biology – functional annotation, imputation, smoothing, etc. There are multiple parameterized approaches out there with little systematic comparison of various approaches and parameters. The authors take on this need and provide a systematic comparative analyses of network diffusion methods and parameters.*

*The work is well motivated, well executed and very well presented. The approaches seem sound. Of the various (matrix normalization) approaches, the results recommend against some of them based on topology bias (the result is biased by the network topology) one is still left with very little idea of which parameters to use for a specific application. Although this work is an honest exploration of a genuine challenge, I have several comments (hopefully to strengthen the work):*

We very much thank the Reviewer for this positive assessment.

*1. Page 11: There are 6 young and 24 old mice. Why only 3 were used for inter-sample variation analysis?*

We think there is a misunderstanding between the age of the mice and the number of replicates and we are sorry about that. The numbers mentioned by the reviewer correspond to the age of the mice (young mice are 6 months old while old mice are 24 months old). However, the number of replicates/samples is 6: 3 young and 3 old mice. All 6 replicates have been used both in the MSE and inter-replicate analyses. We have tried to make this more explicit (see p. 10).

*2. Page 11: Authors have presented MSE across genes with various spreading parameter. It would be more informative to look at the distribution of errors across genes, and not just an overall MSE. And if there is a large variability in error for genes, what characterizes the genes with higher error v low error (something to do with network topology perhaps)?*

This is an excellent thought that we have implemented in our analysis! It had lead to the creation of an entirely new figure (new Fig. 3) and additional insights into the effects of network propagation (see also p. 12, 26).

*3. Page 12: For the inter-replicate consistency, the improvement is minimal at best and in some cases achieved at alpha=1, which is not very useful. What is a biologist supposed to make of this? Some considered discussion is needed at the minimum.*

We have a respective statement in Methods explaining why the correlation value at alpha= 1 (or very large *t*) is not (biologically) meaningful. We agree with the Reviewer that the choice of optimal value for the spreading parameter based on the Pearson correlation (inter-replicate consistency) as a criterion seems less clear compared to the MSE on the datasets tested here. These examples demonstrate that the different optimization criteria do not yield identical results. We have clarified in the Discussion section (see p. 26) this limitation (also addressing Comment 5 of Reviewer #1) stating:

“On the datasets tested here, using the MSE (first criterion) resulted in a more distinct optimum for the spreading parameters than using the correlation-based measures (second and third criterion).”

We also refer to our reply to point 6 below, which is related to this point.

*4. Fig 5A-D: This is nice, but please quantify what fraction of such genes/proteins with missing value were deemed significantly differential based on imputed value.*

In this case the node scores were the fold changes using the average across all replicates. Thus, also the imputed values are fold changes and it is not possible to determine the significance of individual effects (of individual proteins or transcripts). However, the median absolute fold changes of known ageing-associated genes were greater than for the background in all cases that we tested and they were statistically significant in two cases. We have made it more explicit that we are imputing fold changes, not expression values:

“In order to test this idea and in order to further validate the plausibility of network propagation results, we imputed expression fold changes (young versus old) for missing proteins and mRNAs with the goal to recover known ageing-associated proteins and transcripts.”

*5. Fig 5E: Is it correct that ONLY 4 genes are differential after multiple testing correction? Any comments on this? If so, is this even a good data to be analyzing?*

We have checked that these are the numbers of DE genes after multiple testing correction. One reason explaining the low number might be that very few of the genes are highly significant (i.e. with p-value < $10^{-6}$). We are confident about the quality of this dataset though since both quality control and analyses have yielded biologically meaningful results that we were able to validate in independent prostate cancer (PCa) cohorts. Regarding the choice of the data: the application of network propagation on ‘easy’ datasets with a very strong signal may be less informative in this context. Here, we wanted to explore a potential benefit of network propagation for de-noising ‘difficult’ data.

*6. Overall, the results are quite mixed in terms of showing the value of network propagation. Are there range of parameter values that can be recommended that covers the optimal choice across various applications and various optimality criteria? Without this, what is the impact of this work for actual application of these methods?*

Using several datasets we have shown that the utility of network propagation and the optimal parameter value is data-dependent and application-specific: for instance in datasets with large signal-to-noise ratio like the liver rat dataset, network propagation is not expected to help significantly while in the PCa protein dataset more aggressive smoothing was necessary. Hence, smoothing parameters need to be re-calibrated to each dataset. Further, the criteria for choosing optimal parameters will be specific to the research question. For instance, in the presence of multi-omics data it may be desirable to choose the between-dataset consistency among the proposed criteria while the inter-replicate consistency should be selected if it makes sense to have as similar replicates as possible. If one would like several criteria to be met at the same time then one possibility is to select the parameter value optimizing a combination (e.g. normalized sum) of the individual criteria. The same principle can be generalized with several applications. We have added a paragraph in the Discussion (see p. 26):

“The different criteria proposed for optimizing the spreading parameters might lead to distinct solutions. In this case, one needs to consider the research question and the context. For example, does one expect

that the different omics layers should be consistent? Is it more important to increase the correlation between replicate measurements or between omics layers? Yet another possibility is to optimize a combination (e.g. a normalized sum) of the previous criteria so that they are met at the same time."

*7. Even though, based on topological bias, which manifests itself only at the extreme levels of alpha value, authors have ruled out certain smoothing approaches (which by the way has been used a lot), it will still be useful to include those methods in the evaluations – it may still be useful for certain applications.*

This comment is related to Comment 1 of Reviewer #1 (see above). We therefore also point to our reply above.

The topology bias will appear as soon as we move away from alpha=t=0 and the network will start to have an effect on the propagated results. Thus, the bias is always present, yet it gets maximal for most aggressive smoothing. We agree with the reviewer that matrix normalizations inducing topology bias have been used a lot in practice and proven to be useful. For instance, in Charmpi et al. 2020 using network propagation with the normalized Laplacian yielded biologically meaningful results which were further validated. And if the genes associated with the disease/phenotype under study happen to be high-degree nodes then smoothing approaches with a topology bias might help to identify them more easily. However, with such approaches there is always the risk of identifying hits only because they have many neighbors although they have no association with the disease under study (false positives). We have added a relevant statement addressing this point in the Discussion (see p. 25).

"Graph normalization techniques inducing a topology bias might still be useful in practice though. However, one should be aware of a potential topology bias and about the impact of the chosen normalization approach on the final result."

*8. Finally, given that there is SO MUCH data out there, the application seems very narrow. I would like to see somewhat broader benchmarking, especially using datasets where the key genes are established.*

So far, we have analyzed two multi-omics datasets - one study on ageing in rat and another study on cancer on humans. Hence, the existing data cover different species, different data types and different research questions. Thus, with this data we were able to address several important aspects of network propagation. For instance network propagation will not be particularly useful for datasets with large signal-to-noise ratios like the liver rat transcriptome data, while more aggressive smoothing might be needed for the protein layer (compared to mRNA). Further, key genes are established for both studies, e.g. we are using an independent list of known ageing associated genes to validate the results (see Fig. 6). We are not quite certain what the additional insight of including yet another dataset would be. However, we did realize that the potential benefit of network propagation for denoising data could be shown in a better way. In order to address this question more rigorously we have performed a new analysis: we have added noise to the real data and analyzed to what extent network propagation was able to recover the original signal (see p. 11-12 and Fig. 2E-F). We hope the Reviewer agrees with us that this additional analysis provides more insight into network propagation than yet another dataset.

*9. Supplementary Fig 3: Y-axis should be labelled 'inter-sample..', not 'within-sample..'*

Here, for each tumor sample we computed the variance of its propagated $log_2$ fold changes across the genes- this is why we use the term "within-sample" variability.

*10. Page 12: wherever correlations are used, please use Spearman correlation to avoid outlier effect, and specify.*

We have been using Pearson correlation so that information is not lost (e.g. when replacing actual values by their ranks). We have also used Spearman correlation to measure the within-dataset and between-dataset consistency (Figs. 4 & 5), and added the results in the Supplement (Supplementary Figs. 6 & 7). Whereas the numbers slightly differ between the two approaches, the general conclusions are not affected by the choice of the correlation measure.
